# Magnetic and Tunable Sound Absorption Properties of an In-Situ Prepared Magnetorheological Foam

**DOI:** 10.3390/ma13245637

**Published:** 2020-12-10

**Authors:** Noor Sahirah Muhazeli, Nur Azmah Nordin, Ubaid Ubaidillah, Saiful Amri Mazlan, Siti Aishah Abdul Aziz, Nurhazimah Nazmi, Iwan Yahya

**Affiliations:** 1 Engineering Materials and Structures (eMast), Malaysia-Japan International Institute of Technology, Universiti Teknologi Malaysia, Jalan Sultan Yahya Petra, Kampung Datuk Keramat, Kuala Lumpur 54100, Malaysia; nsahirahmuhazeli@gmail.com (N.S.M.); amri.kl@utm.my (S.A.M.); aishah118@gmail.com (S.A.A.A.); nurhazimah@utm.my (N.N.); 2 Mechanical Engineering Department, Universitas Sebelas Maret, J1. Ir. Sutami 36A, Kentingan, Surakarta 57126, Indonesia; 3 International Center, Tokyo City University, 1 Chome-28-1 Tamazutsumi, Setagaya, Tokyo 158-0087, Japan; 4 Department of Physics, Universitas Sebelas Maret, J1. Ir. Sutami 36A, Kentingan, Surakarta 57126, Indonesia; iyahya@mipa.uns.ac.id

**Keywords:** magnetorheological materials, magnetic foam, magnetic saturation, in situ, sound absorption

## Abstract

Conventional polyurethane foam has non-tunable sound absorption properties. Here, a magneto-induced foam, called magnetorheological (MR) foam, was fabricated with the feature of being able to tune sound absorption properties, primarily from the middle- to higher-frequency ranges. Three different samples of MR foams were fabricated in situ by varying the concentration of Carbonyl Iron Particles (CIPs) (0, 35, and 75 wt.%). The magnetization properties and tunable sound absorption characteristics were evaluated. From the magnetic saturation properties, the results showed very narrow and small coercivity of hysteresis loops relative to the soft magnetic properties of the CIPs. MR foam with 75 wt.% CIPs showed a higher magnetic saturation at 91.350 emu/g compared to MR foam with 35 wt.% CIPs at 63.896 emu/g. For tunable sound absorption testing, the effect of ‘shifting’ to higher frequency was also observed when the magnetic field was applied, which was ~10 Hz for MR foam with 35 wt.% CIPs and ~130 Hz for MR foam with 75 wt.% CIPs. As the latest evolution of semi-active noise control materials, the results from this study are valuable guidance for the advancement of MR-based devices.

## 1. Introduction

Sound-absorbing materials are defined as having the ability to absorb the energy of sound waves as much as possible while minimizing reflection and transmitting the energy of the sound waves at the same time [1,2]. A material that can absorb and relay more sound waves than it reflects is considered a good sound-absorbing material. A few requirements of suitable sound-absorbing materials may need to be considered in order to minimize the noise generated; examples include porosity, weight, absorption ability, and range of frequency absorption [3,4]. These sound-absorbing materials are classified into three main groups: passive, active, and semi-active noise control materials [5,6]. These materials could control mechanisms during the sound absorption phenomenon [7,8].

Passive noise control materials can be defined as passively minimizing radiated noises by energy absorption [6,9,10]. This material involves a method that does not require an external supply of control energy by dissipating the propagation of the acoustic waves through various damping mechanisms [1]. Some of the examples of passive noise control materials are foams [1], felt [11], fibers [12], and glass wool [5,7,13], and they have been widely used in the context of building and transport applications [13]. However, the efficiency and sound absorption performance of passive noise control materials are poorer at lower-frequency ranges (20–200 Hz) [14], compared to that of active materials, for which extra energy consumption is inevitable [6,13,15]. Some challenges in using passive noise control materials are primarily related to the long wavelength of sound at low-frequency levels, which results in poor sound absorption [16].

Consequently, active noise control materials have been developed to enhance noise reduction at low-frequency levels (20–200 Hz) [3,17,18]. Nevertheless, from the middle- to high-frequency range (from 200 Hz to 6 kHz), active noise control materials are difficult to implement due to the difference in the phenomenon of sound propagation [6]. This challenge can be explained by the difference in ability to control the propagation of sound waves in active noise control materials, which is achieved by integrating the interference from the propagation of sound waves. In order to perform destructive interference, the sound waves need to integrate with the source wave that is generated between the middle- to high-frequency of ranges of sound absorption in different ways [19]. In addition, even though the introduction of air gaps in the impedance tube has significantly improved lower-frequency sound absorption during testing [20], passive and active noise control materials only absorb sound at one single frequency. Besides, semi-active materials have the potential to absorb sound in a controllable range of frequencies by manipulating the magnetic field values instead of increasing the air gap or the thickness of the materials [21]. In this regard, the limitations of passive and active noise control materials have contributed to the development of semi-active noise control materials.

Semi-active noise control materials can regulate and achieve a compromise between active and passive control systems, accomplished by combining the inherent reliability of passive systems and the adaptability of active systems and requiring significant sources of external power [22]. Besides, semi-active noise control materials are inherently stable [23] compared to passive and active noise control materials. The sound characteristics of the materials can be changed in a desirable manner (broad range of sound absorption frequencies) with semi-active tunable properties over a controllable range of frequencies [23]. In recent years, few attempts have been made to develop semi-active noise control materials by utilizing filler particles; this step caters to the middle- to high-frequency range (from 200 Hz to 6 kHz) of sound absorption [24,25]. Magnetic fillers such as carbonyl iron particles (CIPs) that are reinforced in an absorbent foam matrix are a great candidate to provide a semi-active attenuation material; these particles manipulate magnetic field sources during tunable sound absorption characterization [3,21].

Magnetorheological (MR) foam has been recently developed due to its high potential to become a controllable sound absorber material in tactile devices [26]. This smart foam is believed to control noise over a broad range of frequency absorption bands, ranging from middle to high frequencies (from 200 Hz to 6 kHz), depending on the values of the given magnetic field. A few studies investigated the effects of the magnetic field on the sound absorption of polymer foam coated with MR fluid. In a study carried out by Zielinski and Rak [21], foam coated with MR fluid (MR fluid foam) and exposed to an applied magnetic field showed a significant improvement in the sound absorption coefficient (SAC) at higher frequency, which was above 3.8 kHz. The results also showed a shift to a higher frequency range of about 400–500 Hz, with a magnetic field measurement of 1.25 T. From the study, the SAC of MR foam exposed to a magnetic field could be increased up to 25% compared to the foam without CIPs. However, Zielinski and co-authors only focused on foam prepared via an ex situ method and only on one condition of the magnetic field, which was at 1.25 T. Scarpa et al. [10,27] stated that the presence of two different permanent magnets (sources of a magnetic field), neodymium (1.25 T) and ceramic (0.25 T), had altered the location of the sound absorption frequency of an auxetic foam with a shift from 1.8 to 2.2 kHz. It was specified that a significant shift of ~400 Hz to a higher-frequency range had provided an early indication for the possible use of auxetic foam as a medium in noise control applications [10].

However, in previous literature [10,21,27], fundamental information on MR foam materials fabricated in situ method has been limited, and no detailed explanations on the relationship between the shifting frequencies and tunable sound absorption properties have been reported. Plus, previous studies only reported on magnetic foams that were fabricated via ex situ processes [21,27], whereas in the present study, MR foam materials were fabricated via an in situ method. In situ MR foam production involves internally combining magnetic particles with an absorbent matrix medium, whereas the ex situ method involves externally incorporating the mixture of magnetic particles and suspension medium (such as fluid, elastomer, or plastomer) into the ready foam [3,28]. Compared to the ex situ method, which requires additional manufacturing steps and creates an issue of agglomerated magnetic particles in a porous, absorbent polymeric foam matrix, in situ fabrication contributes to the homogeneous distribution of magnetic particles and creates stronger bonding between magnetic particles and the porous, absorbent MR foam matrix. This homogeneous distribution of magnetic particles in the foam will contribute to the large dispersion of sound waves and to the wide range of frequencies.

In this study, a semi-active MR foam was proposed as a smart magnetic foam that has controllable sound absorption properties, ranging from middle to high frequencies (from 200 Hz to 6 kHz). Compared to conventional sound absorbers that can only absorb noises under certain conditions or one single frequency, this semi-active material is indeed superior, with interesting properties that can manipulate the level of sound absorption accordingly with our requirement. The ability to manipulate sound absorption using a semi-active control was achieved by applying an external magnetic field during tunable characterization [3,24]. Thus, the tunable sound absorption of MR foam in various on-state conditions (0.25 T and 0.50 T), and with different concentration of CIPs (0, 35, and 75 wt.%) embedded into the matrix via in situ fabrication, would strongly influence the magnetic and sound absorption properties. To the best of the authors’ knowledge, it is essential to explore the potential of MR foam as a smart sound absorber over a tunable and wider range of sound absorption, which directly relates to the capability of this MR foam as a promising semi-active material, controllable sound absorber, smart sensor, and tactile device. Other interesting potential applications of MR-based devices can be investigated in the future.

## 2. Materials and Methods

Carbonyl iron particles (CIPs), which are derived from the decomposition of pentacarbonyl iron, were used as magnetic particles and were purchased from CK Materials Laboratory Co. Ltd., Seoul, Korea (http://ckmaterialslab.com/company/) with a diameter between 3 µm and 5 µm. The matrix-based MR foam was developed using a combination of rigid polyurethane (PU) foam containing a polyether polyol (RG135NFDH1) reactant purchased from PPT (M) Sdn. Bhd., Kuala Lumpur, Malaysia, and polymethylene polyphenyl polyisocyanates (4,4′-diphenylmethane diisocyanate (4,4′-MDI) purchased from Tosoh Corporation, Tokyo, Japan. Detailed information on the components used in the fabrication of MR foam is tabulated in Table 1.

In this study, MR foam samples were fabricated in situ by internally mixing the magnetic particles with absorbent matrix components [29]. CIPs are the reinforcement filler, while the combination of polyether polyol (RG135NFDH1) and 4,4′-diphenylmethane diisocyanate (4,4′-MDI) components is the absorbent foam matrix of the MR foam. The total mass of the absorbent matrix components (RG135NFDH1 reactant, 4,4′-MDI, and water) was constant at 30 g, while the specific total mass of CIPs was added to the mixture of foam. The quantity of CIPs and the total mass of the absorbent foam matrix components of the virgin foam and MR foam samples are tabulated in Table 2.

**Figure 1:**
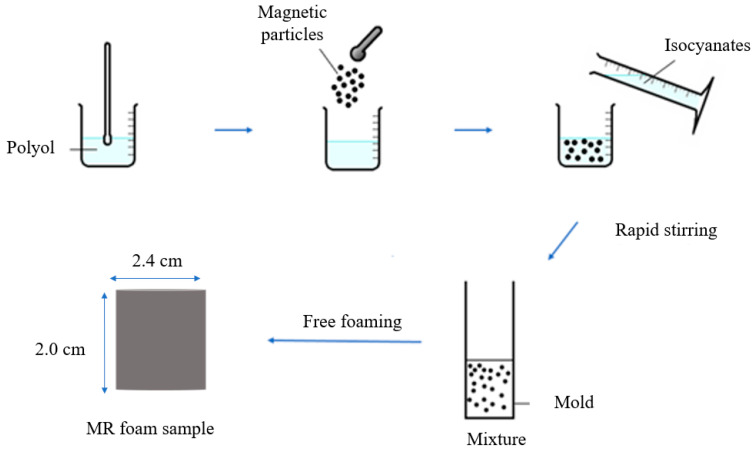
In situ fabrication of MR foam.

The weight percentage (wt.%) term was used, as the samples were produced using CIPs (solids) dissolved in the components of the absorbent matrix. Three different concentrations of CIPs, 0 g (0 wt.%), 10.5 g (35 wt.%), and 22.5 g (75 wt.%), were characterized to study the effect of CIP addition on the properties of MR foams. Figure 1 shows an illustration of the in situ fabrication method that was carried out to produce MR foams. The samples were produced by stirring the polyether polyol reactants for 10 min using a mechanical stirrer at a speed of 350 rpm until the color of the reactants changed from light brown to white. Then, different CIP contents (0 g, 10.5 g, and 22.5 g) were added separately to the reactants, and the mixture was stirred at a speed of 350 rpm for another 5 min. 4,4′-diphenylmethane diisocyanate (4,4′-MDI) and water were added to the mixtures afterward and continuously stirred for an additional 10 s. A cream time of around 18 s was recorded, and at the same time the liquid mixtures began to expand and transform to solid foam within 2–3 min. The MR foams were then left at room temperature for 24 h to ensure the mixture was completely cured.

## 3. Characterization

### 3.1. Magnetic Properties

In this study, the magnetic properties of MR foams embedded with CIPs were analyzed using a Vibrating Sample Magnetometer (VSM), Micro Sense, EZ series, Lowell, MA, USA. Magnetic saturation was measured in a magnetic field ranging from −15 kA/m to 15 kA/m. The importance of measuring the magnetic properties was to determine the parameters or the magnetic behaviors of the particles used, such as the saturation, retentivity, and remanence and coercive force. Each of the sample tests was repeated three times in order to reassure the consistency of values, and the average values of magnetic properties were obtained.

### 3.2. Sound Absorption

**Figure 2:**
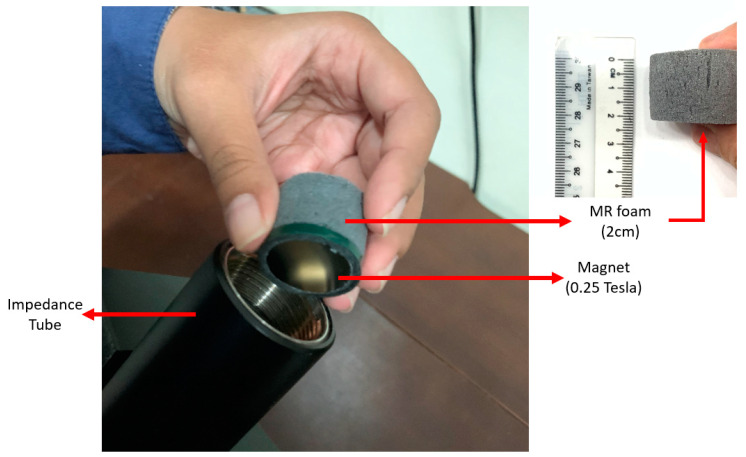
Tunable sound absorption setup.

**Figure 3:**
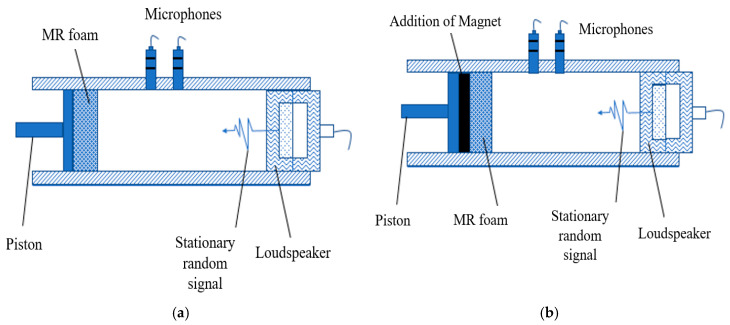
Schematic diagram of (**a**) off-state and (**b**) on-state set-up for acoustic absorption testing.

The general purposes of testing sound absorption were to measure the tunable sound absorption frequencies and coefficient of MR foams and to investigate the effect of the applied magnetic field (on-state) during testing. The sound absorption properties of MR foams were measured using the B&K 4206-T Impedance Tube (Brüel & Kjær, Sound & Vibration Measurement, Nærum, Denmark) based on ISO 10534-2 and ASTM E1050-98. The sample dimensions were 2.0 cm in thickness and 2.4 cm in diameter (Figure 2), and samples were tested in the frequency range from 0 Hz to 6500 Hz, with and without the presence of a magnetic field (using permanent magnets (PMs) with neodymium types); 1 PM (0.25 Tesla, on-state (1)) and 2 PM (0.50 Tesla, on-state (2)). Each of the sample tests was repeated three times in order to reassure the consistency of the values, and the average values of magnetic properties were obtained. Figure 3 shows a schematic diagram of the sound absorption characterizations that were carried out in off-state and on-state conditions. Figure 3a,b illustrate the off-state and on-state acoustic impedance tube set-up, respectively, in which a magnetic field was applied by the addition of a permanent magnet (PM) located after the MR foam in the impedance tube. The addition of PM in Figure 3b created interference, which leads to the potential ability and effectiveness of using MR foam as a tunable sound absorber.

**Figure 4:**
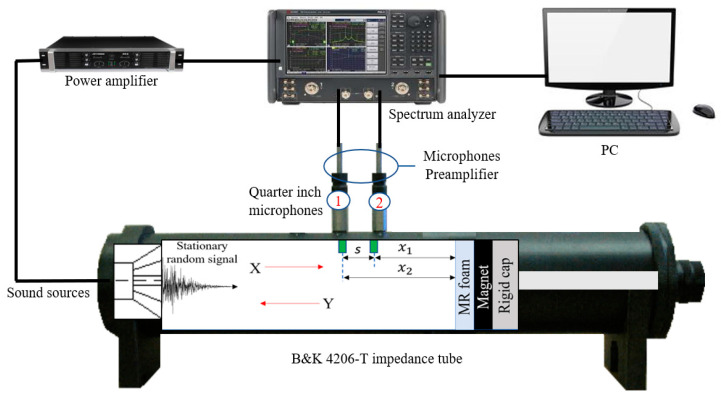
Schematic diagram of sound absorption testing and measurement of absorption coefficient of MR foam.

Figure 4 shows a schematic diagram of how we tested sound absorption and measured the absorption coefficient of MR foam. As shown in Figure 4, the B&K 4206-T impedance tube was equipped with an internal fixed loudspeaker, and two microphones were placed at opposite ends of the loudspeaker. A loudspeaker was mounted in an upstream tube to generate white noise, as driven by a spectrum analyzer (B&K 3160-A-042) and amplified with a power amplifier (B&K 2716C). To capture the processed sound, the microphone preamplifier (B&K2670) was connected to quarter-inch microphones (B&K 4187), and the results were obtained using B&K 7758 acoustic material testing software. The propagation of sound waves in the impedance tube began with a stationary, random signal generated by the loudspeaker [21]. Then, the generation of incident waves *x*(*t*) followed, which propagated to the test sample. As the waves hit the sample surface, a portion of the waves was reflected as *y*(*t*), while the other portion penetrated the test sample and the energy absorbed. Since the tube has a rigid end, no portion of the sound wave was transmitted. Thus, only incident and reflected waves exist in the system. Therefore, based on two microphones transferring impedance functions in the tube, the input–output relation is given by:

(1) y(t)=h(t)×x(t)

where *h*(*t*) is the response of the system. By using the convolution theorem, Equation (1) becomes

(2) $$Y\left(f\right)=H_{12}\left(f\right)\times X\left(f\right)$$

Or:

(3) $$H_{12}\left(f\right)=\frac{Y\left(f\right)}{X\left(f\right)}$$

where $H_{12}\left(f\right)$ is the transfer function, X(f) and Y(f) are the spectra of the incident and reflected waves, respectively, defined by Fourier integrals as follows:

(4) $$X\left(f\right)=\int_{-\infty }^{\infty }x\left(t\right)e^{-j2\pi ft}dt$$

(5) $$Y\left(f\right)=\int_{-\infty }^{\infty }y\left(t\right)e^{-j2\pi ft}dt$$

where $j=\sqrt{-1}$. The transfer function between two microphones $H_{12}\left(f\right)$ can be specified as in Equation (6) [30,31]:

(6) $$H_{12}=\frac{Y}{X}=\frac{e^{jkx_{1}}+Re^{-jkx_{1}}}{e^{jk\left(x_{1}+s\right)}+Re^{-jk\left(x_{1}+s\right)}}$$

Whereas *s* is the spacing between two microphones, and *x* is the distance of microphones from the sample. The measured reflection coefficient (*R*) is expressed in Equation (7), where *k* is the wavenumber [32]:

(7) $$R=\frac{H_{12}-e^{-jks}}{e^{jks}-H_{12}}\;e^{j2k\left(x_{1}+s\right)}$$

The normal incidence sound absorption coefficient (α) can be calculated using Equation (8):

(8) $$\alpha =1-{\left|R\right|}^{2}$$

Whereas the normalized impedance ratio can be obtained from Equation (9):

(9) $$\frac{z}{pc}=\;\frac{1+R}{1-R}$$

## 4. Results and Discussion

### 4.1. Magnetic Properties

**Figure 5:**
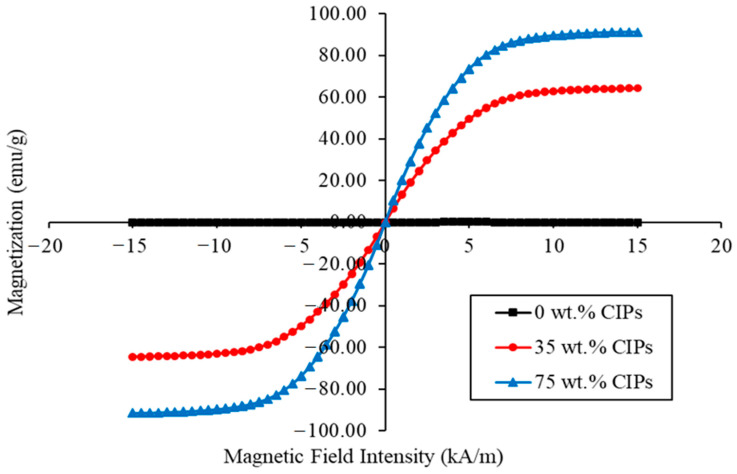
Magnetization curve of MR foam with different concentrations of CIPs.

Magnetic analysis is one of the significant ways to determine the magnetic behavior of particles (CIPs) used in the fabrication of MR foams. Information such as the magnetization saturation (*M_s_*), remanence (*M_r_*), and coercivity (*H_c_*) were obtained from the magnetization curve of the materials, also known as the hysteresis loop of the materials [33]. These values are necessary to analyze the micromovement of the CIPs in each of the MR foam samples so that the magnetic dipole characteristics can be determined. In this study, the magnetic properties of the MR foams and the control sample were analyzed using VSM at a maximum field of 15 kA/m. Figure 5 illustrates the magnetic hysteresis loop of MR foams with different concentrations of CIPs. Table 3 presents the important magnetic parameters obtained from the hysteresis loops such as *M*_s_, *M_r_*, and *H_c_*. As shown in Figure 5, Sample A, which is considered the virgin foam, showed no response towards the applied magnetic field and exhibited a diamagnetic behavior. Furthermore, there was no change in the magnetic saturation (*M_s_*) value. This phenomenon was due to the non-incorporation of CIPs or magnetic particles in the structures of the virgin MR foam.

As for the MR foam samples, Sample C (75 wt.% CIPs) indicated the highest value of *M_s_* at about 91.350 emu/g, as compared to Sample B (35 wt.% CIPs) at 63.896 emu/g. All MR foam samples showed similar trends in the hysteresis loop, which indicated very narrow and small coercivity relevant to the soft magnetic behavior of CIPs embedded in the MR foam samples [18]. Table 3 shows that the MR foams possessed superparamagnetic properties at room temperature, since nearly zero remanences *M_r_* (0.138 emu/g for sample B and 0.216 emu/g for sample C) were observed. As discussed by Riesgo et al. [34], the number of magnetic moments improved when the concentration of CIPs increased. Thus, it is believed that an increased quantity of CIPs enhanced the magnetic properties of the MR foams, as the value of *M_s_* depends on the higher magnetic moment of the CIPs. In addition, the differences in *M_s_*, *H_c_*, and *M_r_* values are believed due to the different contents of CIPs embedded in the MR foam’s absorbent matrices, which influences the magnetization behavior of the materials [3]. It is worthwhile to note that the *M_s_*, *H_c_*, and *M_r_* values were affected by the micromovement of CIPs incorporated in the MR foam [35]. The vibrations of CIPs that occurred in the MR foam matrix were influenced by the magnetic attraction between the struts, which is recognized as a magnetic dipole attraction. The higher the value of *M_s_*, the greater the propagation of CIP micromovement; thus, a stronger magnetic dipole attraction is created, and different dissipation energies of sound absorption are generated.

### 4.2. Tunable Sound Absorption

**Figure 6:**
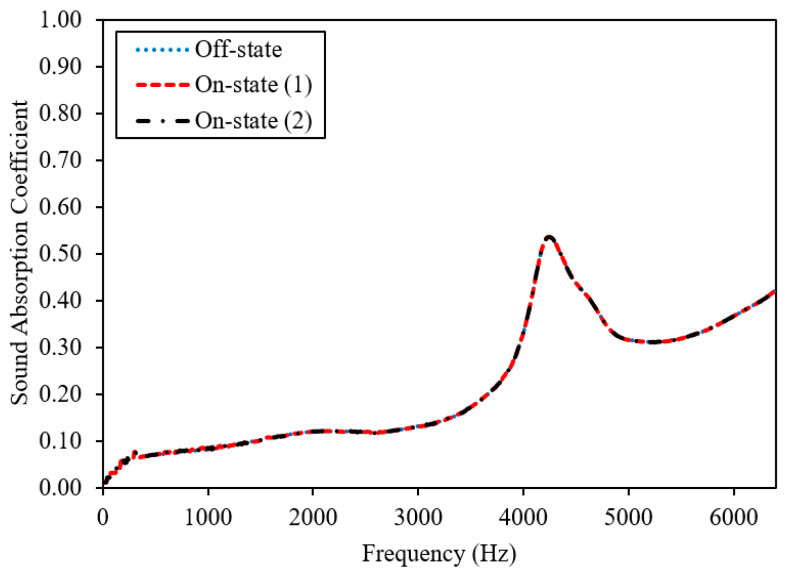
Sound absorption coefficient of virgin foam, 0 wt.% CIPs (Sample A).

**Figure 7:**
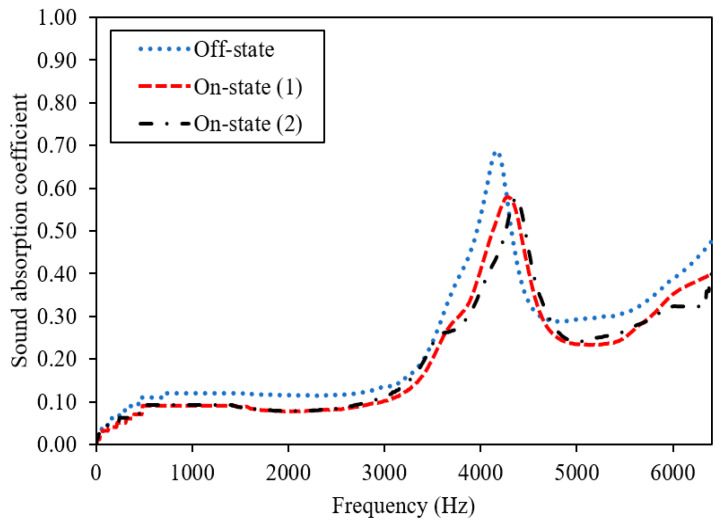
Dynamic sound absorption of MR foam with 35 wt.% CIPs (Sample B).

**Figure 8:**
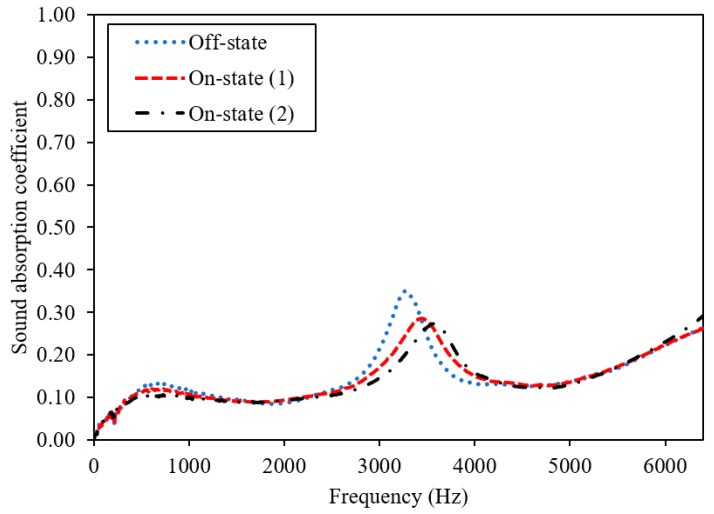
Dynamic sound absorption of MR foam with 75 wt.% CIPs (Sample C).

MR foams possess sound-absorbing or soundproofing properties due to their porous structure and surface density; however, the performance also changes depending on the concentration and orientation of magnetic fillers. Figure 6 displays the sound absorption coefficient of the virgin foam, 0 wt.% CIPs (Sample A), and Figure 7 displays the dynamic sound absorption of MR foams with 35 wt.% CIPs (Sample B). Figure 8 shows the dynamic sound absorption of MR foams with 75 wt.% CIPs (Sample C). Tests were carried out using different values of the magnetic fields (permanent magnets, PM): 1 PM (on-state (1); 0.25 Tesla) and 2 PM (on-state (2); 0.50 Tesla), with diameters of 0.3 cm and 2.4 cm, respectively. A summary of the tunable sound absorption testing of MR foam is presented in Table 4.

The sound absorption of Sample A, the virgin foam without the incorporation of magnetic particles (CIPs), is shown in Figure 6. The highest peak frequency of Sample A was recorded at 4.29 kHz in the off-state condition, and no differences in the frequencies were recorded on-state (1) and on-state (2) conditions, which indicates similar values in the off-state condition. In terms of SAC, there were also no changes in all conditions, representing 0.52 in the off-state, on-state (1), and on-state (2). This phenomenon was predicted since no CIPs were incorporated in Sample A, and the frequencies and the SAC did not change during testing with or without the application of a magnetic field. Meanwhile, as shown in Figure 7, in the off-state condition or without the applied magnetic field, the highest peak frequency of Sample B was indicated at 4.12 kHz, compared to the on-state (1) condition where the absorption peak frequency was 4.33 kHz and the on-state (2) condition where the absorption peak of frequency was 4.34 kHz. This finding indicates that the addition of PM (at on-state (1)) had shifted the absorption of Sample B (35 wt.% CIPs) to a higher frequency, which was from 4.12 kHz (off-state) to 4.33 kHz (on-state (1)) of the sound absorption frequency. Then, when more PM was added, denoted as on-state (2), there was another slight shift in the range of sound absorption frequency of about ~10 Hz, which was from 4.33 kHz (on-state (1) to 4.34 kHz (on-state (2)).

In terms of the peak absorption frequency, the same trend was shown for Sample C (75 wt.% CIPs), which is displayed in Figure 8. In the off-state condition, the highest peak frequency of Sample C was noted at 3.28 kHz, compared to the on-state (1) absorption peak frequency at 3.42 kHz and on-state (2) at 3.55 kHz. This finding indicates that the addition of PM (on-state (1)) shifted the absorption of Sample C (75 wt.% CIPs) to a higher frequency, which was from 3.28 kHz (off-state) to 3.42 kHz (on-state (1)). Then, when another PM was added to the test (on-state (2)), there was another shift of about ~130 Hz in the frequency absorption, from 3.42 kHz (on-state (1)) to 3.55 kHz (on-state (2)). Such effects on the shifted frequency values, as visualized in Figure 7 and Figure 8, are significant because the peak shift in sound absorption can usually be achieved once the magnetic particles in the foam structures respond to the applied magnetic field.

These results also indicate that MR foams can be potentially used as a candidate of tunable sound absorption. A similar shifting effect was proven by Scarpa et al. (2004), who demonstrated the presence of PM had shifted the acoustic absorption peak of auxetic MR fluid foams [27]. They investigated the effects of two different types of PM with different magnetic intensities, neodymium (1.2 T) and ceramic (0.25 T); slightly altered frequencies and decreased values of SAC were found. From the study, the higher intensities of PM had contributed to the higher shift in the peak frequencies of sound absorption. A study by Zielinski et al. [21] showed that there were changes in the sound absorption peak, in which the frequency of acoustic absorption was altered by ~400–500 Hz, to a higher frequency when PM with a magnetic field intensity of 1.25 T was used. Consequently, MR foam is a rigid-framed sound-absorbing material that consists of closed voids or connected pores, which have been intentionally fabricated for sound absorption purposes [3,18]. For foams with closed pores, most of the incident sound waves are reflected, and others will pass through the pores of MR foam. In this state, the vibration energy consumption and Helmholtz resonance will be the main sound absorption mechanisms [36]. Thus, when a certain sound wave passes through the pores of MR foam, the sound energy that propagates in the interconnected voids is dissipated via conversion to heat. This conversion is facilitated by wave refraction and interference inside the structures of the pores. In this study, when a magnetic field was applied to the MR foam, it triggered the CIPs, and more interference from sound energy occurred inside the void’s texture. In this context, interference, which is related to the changes in the stiffness, has occurred due to the response of MR foams towards the magnetic field intensities that were applied during sound absorption testing. Changes in the pore structures of the MR foam were also influenced by the addition of permanent magnets (PMs), which triggered a shift of the peak frequency to a higher range of sound absorption.

**Figure 9:**
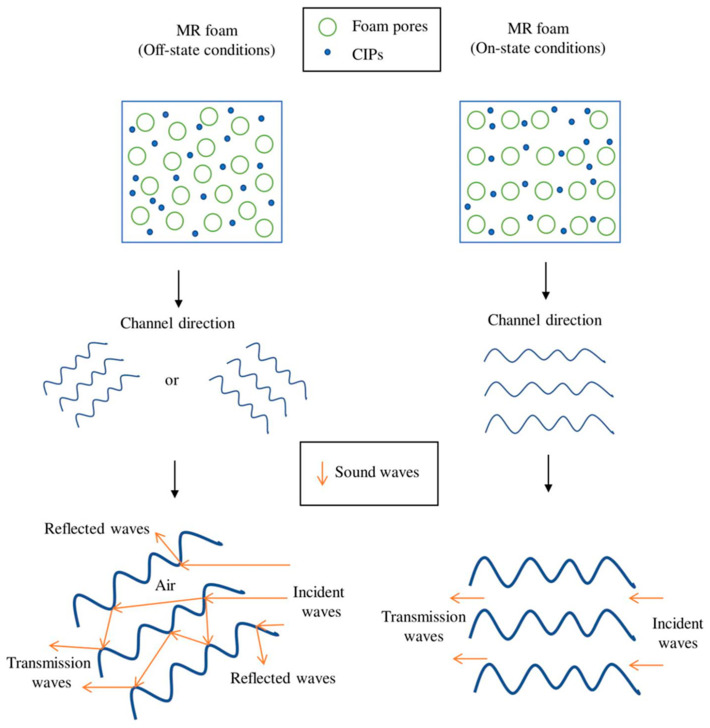
Schematic illustration of the acoustic wave channels in MR foam structures with and without the presence of a magnetic field.

Comparing the absorption pattern frequencies, Sample C exhibited a sound absorption peak over a broader frequency range compared to Sample B. This can be correlated to the different concentrations of CIPs incorporated into each sample, which contributed to the changes in the foam structures. As reported by Muhazeli et al. [3], the size of the foam pores decreased due to the increasing quantity of CIPs incorporated into the cell structures of MR foam. Besides that, in terms of sound absorption coefficient (SAC) values, the addition of a magnetic field using PM contributed to a decrease in SAC values for both Samples B and C. Sample B showed a decreasing trend for the SAC value, from off-state at 0.676 to on-state (1) at 0.569 and on-state (2) at 0.572. Sample C also indicated a decrease in SAC values from 0.350 (off-state) to on-state (1) at 0.286 and on-state (2) at 0.272. Even though the addition of a magnetic field resulted in decreased SAC values, the peak shift to a higher frequency is significant, as this indicates the promising properties of MR foam as tunable sound absorber material. A similar outcome was also reported by Scarpa et al. [27]. From their study, the application of a magnetic field decreased the SAC value from 0.94 to 0.89. This phenomenon can be correlated to the coinciding sound waves that occur due to the application of a magnetic field, which created different parallel channels, as illustrated in Figure 9 [37].

The ability of the materials to absorb sound waves (incident waves) and minimize the reflection and transmission is considered good sound absorption characteristics [1]. Figure 9 shows the behavior of the incident wave channel that strikes the MR foam structure in off-state (without the application of a magnetic field) and on-state conditions (with the application of a magnetic field). Based on the schematic illustration, the sound wave channels in the off-state condition traveled farther and contributed to multiple reflections, and the friction of the airflow consumed more sound energy. However, when a magnetic field was applied (on-state conditions), the direction of sound waves was assumed to become parallel to the magnetic field direction; this contributes to the ease with which incident waves passed through the MR foam structures and contributed to a resonant state, which rapidly lowered the energy of SAC after a specific frequency band [37]. There are at least two basic principles to explain the shift in sound absorption patterns. First, when the magnetic field was off (off-state condition), the elastic parameters and pore size of the MR foam had not changed. The addition of CIP filler triggered the viscous damping mechanism to be more effective. Thus, the absorption impact became more effective. When the magnetic field was activated, the CIPs embedded in MR foam structures were affected so that the CIPs attracted to form a relatively regular formation. As a result of this event, the stiffness of MR foam was altered, thus leading to changes in the pore sizes and material reactance. When the stiffness of the MR foam changed, the pore surface of MR foam structures did not oscillate as it did in the off-state condition. As a consequence, viscous attenuation was not as efficient. The pull of the magnetic field also decreased the pore sizes relative to the off-state condition. Nevertheless, the impact of absorption shifted the peak absorption to a relatively higher frequency.

**Figure 10:**
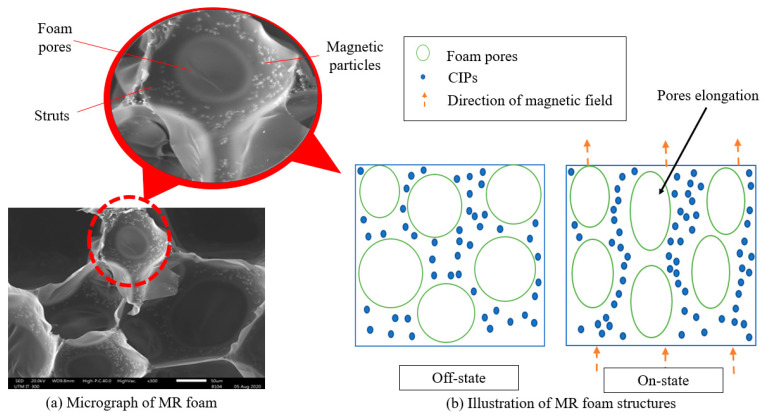
(**a**) Variable Pressure Scanning Electron Micrograph (VP-SEM) of MR foam. (**b**) Schematic illustration of MR foam during on-state conditions.

Figure 10a shows the Variable Pressure Scanning Electron Micrograph (VP-SEM) of MR foam with 75 wt.% CIPs, while Figure 10b shows a schematic illustration of tunable sound absorption during the on-state conditions. From Figure 10a and the close-up micrograph, the round-like particles in the foam struts were assumed as the CIPs that were embedded during the ‘in situ’ fabrication process. Therefore, from the micrographs, it was believed that the CIPs were surely embedded into the struts of MR foam pores. Referring to Figure 10b, when the increased energy of the sound waves that struck the material changed to heat energy, the CIPs could be recognized as individual particles carrying a small reserve of heat. In addition, as specified by Muhazeli et al. [3], CIPs could also act as active reflectors. Thus, when the sound waves struck the MR foams’ structure under the application of a magnetic field, it caused the CIPs to reflect the waves and vibrate more rapidly. At one stage, this vibration caused a lower amount of heat due to friction and sound absorption, which was accomplished by way of heat energy conversion. When sound is absorbed by MR foam, the sound is forced to oscillate at the frequency of sound waves through the closed-like pores, which contributes to the pore’s elongation (Figure 10b). Sound pumping will occur in the foam films within several seconds of encountering a sound wave [38]. Thus, the films on the outside pore layer create thousands of oscillations that will make it easier for sound waves to propagate [38,39]. Accordingly, even though the SAC energy decreases when a magnetic field is applied at a certain stage, the incorporation of CIPs encourages heterogenous collisions. A shift to higher frequencies is also significant and promotes the dynamic sound-absorbing properties of MR foams, especially for potential use as active sound-absorbing materials.

## 5. Conclusions

Throughout this work, MR foam was proposed as a promising candidate that can smartly manipulate sound levels ranging from middle to higher frequencies. Three different MR foam samples were prepared in situ, in which varying concentrations of CIPs (Sample A (0 wt.% CIPs), B (35 wt.% CIPs), and C (75 wt.% CIPs)) were added into the absorbent matrix. The effects of various CIP contents and different magnetic field conditions (0.25 T and 0.5 T) on the tunable sound absorption and magnetic properties of MR foam have been experimentally investigated and evaluated. From the test, the following results have been obtained.

- From the magnetic properties investigation, it was found that Sample C displayed the highest value of magnetic saturation (*M*_s_), which was about 91.35 emu/g; Sample B was 63.896 emu/g. Conversely, a very narrow and small coercivity was indicated for all MR foam samples, which is related to the soft magnetic behavior of CIPs.
- From the tunable sound absorption testing, the addition of a magnetic field using permanent magnets (PMs; 0.25 T and 0.5 T) shifted the frequency range values of MR foams, which shows this MR foam is a promising semi-active material, especially for use in smart sound-controllable devices. When the magnetic field was off (off-state condition), there was no change in the elastic parameters and pore size of the MR foam. The addition of CIPs as a magnetic filler and the addition of permanent magnets as the source of the magnetic field triggered the effectiveness of MR foam as a promising semi-active material with viscous damping properties. Even though application of a magnetic field using the permanent magnets contributed to decreased SAC values, the peak frequency shift from the middle to higher range, indicated as the so-called ‘shifting effect’, was relatively substantial because it allows one to tune the sound absorption characteristics.

The results presented in this work indicate that MR foam could be a promising candidate for use as a semi-active noise-controllable material. Consequently, the ability to manipulate the sound absorption frequency by varying the magnetic field may have a significant effect on the sound absorption properties and characterization. Even though implementation of this semi-active noise control concept is still new, the results in this work will be useful for the design of MR-based devices.

## Figures and Tables

**Table 1:** Detailed information on the matrix-based components used in MR foam fabrication.

Components Characteristics	Absorbent Foam Matrix	
	Polyol	Isocyanates
Chemical Name	Polyether Polyols (RG135NFDH1)	4,4′-Diphenylmethane Diisocyanate (4,4′-MDI)
Viscosity	200 ± 50 cps	300 ± 50 cps
Cream Time	10 ± 8 s	
Gel Time	150 ± 15 s	
Tack Free	260 ± 20 s	
Free Rise Density	25–27 kg/m^3^	

**Table 2:** Composition of MR foam.

Sample (wt.%)	The Total Mass of Absorbent Foam Matrix Components (g)	Quantity of CIPs (g)	Remark
0	30	0.0	Virgin Foam (Sample A)
35	30	10.5	In-situ MR Foam (Sample B)
75	30	22.5	In-situ MR Foam (Sample C)

**Table 3:** Comparison of magnetic properties of MR foam with different concentrations of CIPs.

Samples	Magnetization, *M_s_* (emu/g)	Coercivity, *H_c_* (kA/m)	Remanence, *M_r_* (emu/g)
Sample A (0 wt.% CIPs)	0.0	0.0	0.0
Sample B (35 wt.% CIPs)	63.896	0.010	0.138
Sample C (75 wt.% CIPs)	91.350	0.010	0.216

**Table 4:** Sound absorption parameters of virgin foam (Sample A) and MR foam (Sample B and C).

Parameter	Sample	Magnetic Field Conditions					
		Off-State		On-State (1)		On-State (2)	
		Mean	* SD	Mean	SD	Mean	SD
The Highest Peak of Frequency Absorption (kHz)	A	4.29	0.057	4.29	0.043	4.29	0.005
	B	4.12	0.057	4.33	0.011	4.34	0.01
	C	3.28	0.056	3.42	0.005	3.55	0.005
Sound Absorption Coefficient (SAC)	A	0.52	0.057	0.52	0.005	0.52	0.005
	B	0.676	0.056	0.569	0.002	0.572	0.0005
	C	0.350	0.057	0.286	0.005	0.272	0.001

* SD = Standard Deviation.
